# Spatial and temporal evolution and influencing factors of tourism eco-efficiency in Fujian province under the target of carbon peak

**DOI:** 10.1038/s41598-023-50468-8

**Published:** 2023-12-27

**Authors:** Yizhen Wu, Anxin Xu, Chao Wang, Yuting Shi

**Affiliations:** 1 Forestry College, Fujian Agriculture and Forestry University, Fuzhou, 350002 China; 2 School of Journalism and Communication, Minjiang University, Fuzhou, 350002 China; 3 Department of Tourism Management, South China University of Technology, Guangzhou, 511442 China; 4 Anxi College of Tea Science (College of Digital Economy), Fujian Agriculture and Forestry University, Quanzhou, 362400 China

**Keywords:** Environmental impact, Ecology

## Abstract

Tourism eco-efficiency is an important indicator to measure the sustainable development of tourism with the core objective of "minimum resource input and environmental damage, maximum economic and social output". This paper explores the spatial and temporal evolution characteristics of tourism eco-efficiency and its influencing factors in Fujian Province from 2011 to 2020 by combining the unexpected output Super-SBM model, gravity center model, standard deviation ellipse, and optimal parameters-based geographical detector model. The results are as follows. Tourism eco-efficiency in Fujian Province showed an overall fluctuating growth trend with an increase of 160.065% during the study period. The coefficient of variation of tourism eco-efficiency tends to fluctuate and decrease, and regional differences narrow. The spatial differentiation is remarkable, and it presents the spatial distribution characteristics of "Xiamen > Nanping > Quanzhou > Fuzhou > Longyan > Ningde > Sanming > Zhangzhou > Putian". The spatial pattern of tourism eco-efficiency is obvious in the direction of "northeast-southwest", and the trajectory of the center of gravity is "southwest-northeast-northwest", with spatial characteristics tending towards agglomeration. Tourism industry structure has the greatest impact on tourism eco-efficiency, followed by education level, urbanization level, technology innovation level, infrastructure investment, and economic development level. This study provides reference suggestions for improving tourism eco-efficiency which helps to promote adequate, balanced, and sustainable development of the regional tourism industry.

## Introduction

The tourism industry is traditionally regarded as a green industry, but the long-term adoption of a rough tourism economic development model, characterized by pursuing a growth rate and consuming a lot of resources, has not paid attention to the rational development and utilization of tourism resources, with negative impacts on the natural environment gradually emerging while promoting economic and social development. Studies have shown that the energy consumption of tourists in tourism transportation, accommodation, and other tourism activities is increasing, and its carbon emissions have accounted for 8% of the total global carbon emissions^1,2^. China attaches great importance to the construction of ecological civilization and promotes the transformation of the tourism industry from high-speed growth to high-quality development. In response to the problems of factor-driven growth, sloppy development, and lagging tourism eco-efficiency in China's tourism industry development, the strategic goal of "strive to reach peak carbon dioxide emissions by 2030 and achieve carbon neutrality by 2060”, to contribute to China's power to cope with the world energy crisis and adapt to and mitigate global climate change.

The concept of eco-efficiency was first proposed by Schaltegger and Sturn in 1990^3^, The basic idea is to obtain maximum economic output with minimum resource consumption and environmental cost^4,5^. Gossling first introduced eco-efficiency to tourism research in 2005, suggesting that tourism eco-efficiency can be expressed by the ratio of tourism revenue to its environmental impact, emphasizing that the tourism industry is not a "smoke-free industry" in the traditional sense^6^. Tourism eco-efficiency is an important indicator to measure the sustainable development of tourism, emphasizing the creation of the highest economic and social benefits with the least input of tourism factors and minimizing the negative impact on the ecological environment, which provides a new theoretical perspective to solve the problems of how to scientifically assess the quality of tourism industry development and promote the sustainable development of tourism industry^7^.

At present, positive progress has been made in related research in this area. In terms of conceptual definition, domestic scholars summarize tourism eco-efficiency as the reduction of unexpected outputs such as carbon emissions and energy consumption in the tourism process, increasing desired outputs such as income, so as to realize the improvement of the quality and efficiency of tourism^8^, Liu et al.^9^ defined it from the perspective of industrial development as the ratio of the value generated by the tourism industry in providing tourism products to the environmental expenditures consumed by the tourism industry in a certain period of time in the whole region. In tourism eco-efficiency measurement, early scholars mainly used the single ratio method^10–12^, indicator system method^13,14^ for quantitative measurement, in view of the limitations of the above methods, scholars gradually used Data Envelopment Analysis^15,16^ to quantitatively measure tourism eco-efficiency. However, the traditional DEA method cannot fully rank the decision units effectively by ignoring the effect of slack variables on the efficiency measures and the fact that unexpected outputs can be generated in the production process. Tone^17^ proposed a non-radial, non-angular SBM model that includes unexpected outputs based on the traditional DEA model. The principles of the super-efficient DEA model are further applied to the SBM model to develop the Super-SBM model considering unexpected outputs, which combines the advantages of the super-efficient DEA model and the SBM model, not only to solve the problems of input–output slackness and unexpected outputs but also to rank the efficiency values.

With the deepening of research, scholars began to explore the temporal and spatial evolution analysis and influencing factors of tourism eco-efficiency. Such as analyzing the spatial pattern of carbon emission efficiency in China's tourism industry and its influencing factors using exploratory spatial data analysis methods and geographically weighted regression models^18^. Exploring the Influencing factors of Tourism Eco-efficiency with Tobit model^19,20^. Existing studies have made a lot of achievements in tourism eco-efficiency measurement and spatio-temporal evolution analysis, but they lack in-depth analysis of the factors affecting tourism eco-efficiency, and the relevant studies seldom quantitatively explain the strong and weak ordering of the influencing factors, meanwhile, the established studies have mostly applied empirics to determine the key issues of spatial data discretization and spatial scale effects in geographical detector^21,22^, and few have used the optimal combination of spatial discretization^23,24^ as a model parameter for geographical detector to reveal the driving factors. In addition, there is a regional imbalance in the research results related to tourism eco-efficiency, and most of the existing research focuses on the national level, especially fewer discussions on the evolution of spatial patterns and influencing factors at the provincial spatial scale, and a lack of research results on economically developed coastal provinces^5,14^. In fact, studying how to improve regional tourism eco-efficiency is of great practical significance for a region to develop targeted tourism environmental protection policies as well as to improve tourism eco-efficiency.

Based on the above, this paper takes the tourism industry of 9 prefecture-level cities in Fujian Province from 2011 to 2020 as the research object. The methods of the unexpected output Super-SBM model, gravity center model, standard deviation ellipse, and optimal parameters-based geographical detector model are synthesized to expand the perspective and content of relevant studies from the theoretical point of view, and to provide decision support for promoting adequate, balanced and sustainable development of tourism industry in Fujian Province in practice.

## Study area

**Figure 1:**
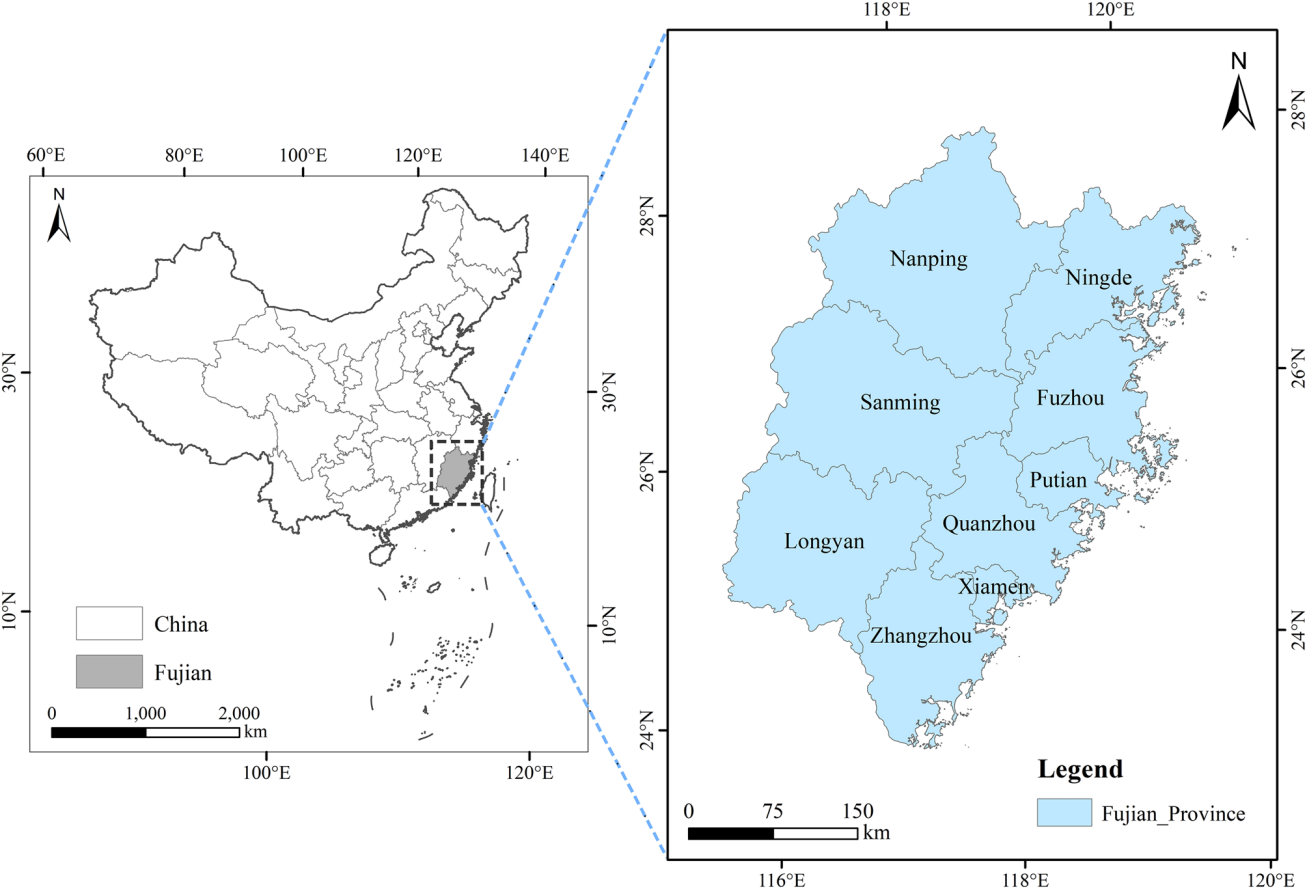
Study area.

Fujian Province is located on the southeast coast of China (Fig. 1), with a land area of 124,000 square kilometers and a sea area of 136,000 square kilometers. It has jurisdiction over 9 prefecture-level cities including Fuzhou, Xiamen, Quanzhou, Zhangzhou, Putian, Longyan, Sanming, Nanping, and Ningde. With rich and unique tourism resources, profound historical and cultural heritage, and early start and mature development of the tourism industry, it is the core area for the development of China's tourism industry as well as the frontier of economic development and opening up to the outside world. The topography of Fujian Province is high in the northwest and low in the southeast, the forest coverage rate is over 80% and has ranked first in the country for 42 consecutive years, the climate is warm and humid, precipitation is abundant, and the construction foundation of ecological civilization is quite good. In 2016, Fujian Province became the first national ecological civilization pilot zone in China, taking the advantage of early and pilot implementation of institutional innovation in ecological civilization construction, accelerating the promotion of green and low-carbon transformation of economic and social development, and making high-level protection of the ecological environment go hand in hand with high-quality economic development. Tourism industry as one of the key kinetic energy to promote the construction of ecological civilization in Fujian Province. In 2020, Fujian Province received 2,296,700 tourists, and realized a total tourism revenue of 507.041 billion yuan, such a huge tourism activities will undoubtedly bring ecological damage to the local area, and increase urban ecological safety risks. However, due to the differences in economic development conditions, resource endowment, industrial foundation and other factors, the problem of regional coordination of economic, social and ecological development in Fujian Province has been prominent for a long time. Based on the micro prefecture-level city perspective, exploring the tourism eco-efficiency evolution law within Fujian Province is of great significance for accelerating the construction of an all-area eco-tourism province and promoting the construction of ecological civilization, as well as providing more replicable and extendable experiences for the national eco-civilization construction.

## Methods

### The unexpected output super-SBM model

Measuring eco-efficiency in Fujian Province using the unexpected output Super-SBM model. The formula is.

1 $$\begin{gathered} \min \rho { = }\frac{{\frac{1}{m}\sum\limits_{i = 1}^{m} {\frac{{\overline{x}_{i} }}{{x_{i0} }}} }}{{\frac{1}{{S_{1} { + }S_{2} }}\left( {\sum\limits_{q = 1}^{{s_{1} }} {\frac{{\overline{y}_{q}^{w} }}{{y_{q0}^{w} }}} + \sum\limits_{q = 1}^{{s_{2} }} {\frac{{\overline{y}_{q}^{b} }}{{y_{q0}^{b} }}} } \right)}} \hfill \\ \overline{x} \ge \sum\limits_{j = 1, \ne k}^{n} {\theta_{j} x_{j} } ,\overline{y}^{w} \le \sum\limits_{j = 1, \ne k}^{n} {\theta_{j} y_{j}^{w} } ,\overline{{y^{d} }} \ge \sum\limits_{j = 1, \ne k}^{n} {\theta_{j} y_{j}^{b} } \hfill \\ \overline{x} \ge x_{0} ,0 \le \overline{y}^{w} \le y_{0}^{w} ,\overline{y}^{b} \ge y_{0}^{b} ,\theta \ge 0 \hfill \\ \end{gathered}$$

In this expression, *ρ* is the measured efficiency value, with higher values indicating higher relative efficiency of the decision unit; *n*, *m*, *S*_*1*_, and *S*_*2*_ respectively denote the number of decision units, input indicators, expected outputs, and unexpected outputs; *x*_*i0*_, *y*^*w*^_*q0*_, and *y*^*b*^_*q0*_ denote input indicators, expected output, and unexpected output indicators, respectively; $\overline{x}_i$, $\overline{y}$^*w*^_*q*_, and $\overline{y}$^*b*^_*q*_ are the slack amounts of the three.

### Gravity center model and standard deviation ellipse

This paper adopts the gravity center model to capture the spatial and temporal evolutionary paths and trends of tourism eco-efficiency. The center of gravity in geography refers to the balance point of a certain attribute value within a certain area. Suppose the region has n sub-regions and the geographic center coordinates of the sub-region is (*X–*_*i*_,*Y–*_*i*_); (*X*, *Y*) are the geographic coordinates of the center of gravity in the area attribute; *P*_*i*_ is the attribute value of the sub-region, which in this study is the tourism eco-efficiency value of each region in Fujian Province.

2 $$X = \mathop \sum \limits_{i = 1}^{n} P_{i} \overline{X}_{i} /\mathop \sum \limits_{i = 1}^{n} P_{i} \quad Y = \mathop \sum \limits_{i = 1}^{n} P_{i} \overline{Y}_{i} /\mathop \sum \limits_{i = 1}^{n} P_{i}$$

On this basis, this paper further adopts the standard deviation ellipse to reveal the spatial and temporal evolutionary characteristics of tourism eco-efficiency in Fujian Province. In a standard deviation ellipse, the trajectory of the center of gravity offset is the trend of the evolution of the spatial pattern reflecting its efficiency; The angle of rotation reflects the direction of the main trend of its distribution; The long and short semi-axes reflect the degree of dispersion of the spatial pattern of their efficiency in the primary and secondary directions, respectively^25^. The formula can be found in the relevant literature^26^.

### Optimal parameters-based geographical detector

Geographic detectors can detect and analyze the spatial differentiation of tourism eco-efficiency as a new statistical method^27^. The optimal parameters-based geographical detector^24^ is an improvement of the geographical detector., which discretizes continuous-type variables. Judging the effectiveness of discretization classification can be evaluated by the *q* statistic of the geographic detector, and the larger the *q*-value the better the partitioning effect^27^. Therefore, with the help of Rstudio software^24^, Using equal spacing classification, natural spacing classification, quantile spacing classification, and standard deviation spacing classification, the discrete mode and discontinuity number with the largest *q* value are screened out. Based on the selection of optimal parameters, factor detection and ecological detection in geographic probes are used to reveal the driving forces of the spatial distribution characteristics of tourism eco-efficiency in Fujian Province.

#### Factor detection

Factor detection can indicate the extent to which influence factors explain the spatial distribution of tourism eco-efficiency, using a *q*-value metric.

3 $$q = 1 - \frac{{\sum\limits_{h = 1}^{L} {N_{h} \sigma_{h}^{2} } }}{{N\sigma^{2} }} = 1 - \frac{SSW}{{SST}}$$

4 $$SSW = \sum\limits_{h = 1}^{L} {N_{h} } \sigma_{h}^{2} ,SST = N\sigma^{2}$$

In this expression, *q* denotes the explanatory power of the factor with a value range of [0, 1], the closer to 1 the greater the explanatory power; *h* is the stratification of explanatory or explained variables; *N*_*h*_ and *N* are the number of units in layer *h* and the whole area; σ^2^_*h*_ and *σ*^2^ represent the variance of layer *h* and whole region *Y* values; *SSW* and *SST* are the sum of within-stratum variance and the total variance of the whole region.

#### Ecological detection

Ecological exploration is used to analyze whether there is a significant difference between the effects of two influencing factors on the spatial distribution of tourism eco-efficiency, as measured by the *F* statistic.

5 $$F = \frac{{N_{X1} \left( {N_{X2} - 1} \right)SSW_{X1} }}{{N_{X2} \left( {N_{X1} - 1} \right)SSW_{X2} }}$$

6 $$SSW_{X1} = \sum\limits_{h = 1}^{L1} {N_{h} } \sigma_{h}^{2} ,\;SSW_{X2} = \sum\limits_{h = 1}^{L2} {N_{h} } \sigma_{h}^{2}$$

In this expression, *N*_*X1*_ and *N*_*X2*_ denote the sample sizes of the two factors *X1* and *X2*; *SSW*_*X1*_ and *SSW*_*X2*_ denote the sum of the within-stratum variance of the stratum formed by *X1* and *X2*; *L1* and *L2* represent the number of variable *X1* and *X2* stratifications. where the null hypothesis *H*_*0*_: *SSW*_*X1*_ = *SSW*_*X2*_. If *H*_*0*_ is rejected at the significance level of *α*, it indicates that there is a significant difference in the effect of the two factors *X1* and *X2* on the spatial distribution of attribute *Y*.

## Indicator selection and data source

### Indicator selection

Tourism eco-efficiency is a process evaluation of tourism activities, and the selection of indicators is a key issue in the assessment, the evaluation indicators are input indicators and output indicators. Drawing on previous research, this paper selects six indicators to build the evaluation index system of tourism eco-efficiency, as shown in Table 1. In terms of tourism eco-efficiency input indicators, land, labor, and capital are the most basic production input factors in economic activities, and tourism activities cannot be separated from the factor of land, however, it is difficult to define and count the scale of land input in tourism activities, and relevant studies rarely include this indicator, so this paper does not include land factor input. Tourism workers are the most intuitive criterion reflecting the labor factor, highlighting the labor-intensive characteristics of the tourism industry^8^, and selecting tourism workers as labor input; The investment of capital elements ensures the smooth development of tourism activities, but at present , most cities do not have official statistics on the indicator of the amount of fixed asset investment in the tourism industry; therefore, the amount of fixed asset investment in the tertiary industry is selected to characterize capital investment, although it amplifies the scale of the actual capital element investment in the tourism industry to a certain extent, it can highlight the comprehensive characteristics of the diversified and diverse tourism industry^28–30^; The energy consumed during tourism activities is also an important indicator of its input^31,32^. In terms of tourism eco-efficiency output indicators, the expected output is expressed in terms of total tourism revenue and the total number of tourists selected at the level of economic efficiency and market size, which are the most direct products of tourism activities and the output indicators most often chosen by scholars to measure the efficiency of the tourism industry^33^. The unexpected output is expressed using carbon emissions, which is a mature method and involves a wide range of pollution^10^.

**Table 1:** Tourism eco-efficiency indicators.

Indicators	Category	Specific indicator
Input indicator	Labor input	Tourism workers
	Capital investment	Investment in fixed assets of tertiary industry
	Energy input	Tourism energy consumption
Output indicator	Expected output	Total tourism revenue
		Total number of tourists
	Unexpected output	Tourism carbon emissions

### Data source

This paper takes the “Twelfth Five-Year Plan” and “Thirteenth Five-Year Plan” as the research period, that is, 2011–2020. During this period, tourism accelerated its integration into the national strategic system, was in the stage of reform and development, and gradually became an important driving force for economic transformation and upgrading as well as ecological civilization construction. Considering the reliability of the data fully, the input and output panel data sets are mainly derived from the Fujian Provincial Statistical Yearbook, the Fujian Provincial National Economic and Social Development Bulletin, the Statistical Yearbook of each city, and the National Economic and Social Development Bulletin of each city. Tourism energy consumption and tourism carbon emissions are measured by the "bottom-up" method of Shi Peihua^34^, with tourism transportation, tourism accommodation, and tourism activities as the main sources of carbon emissions^35^.

## Results

### Temporal characteristics of tourism eco-efficiency

**Figure 2:**
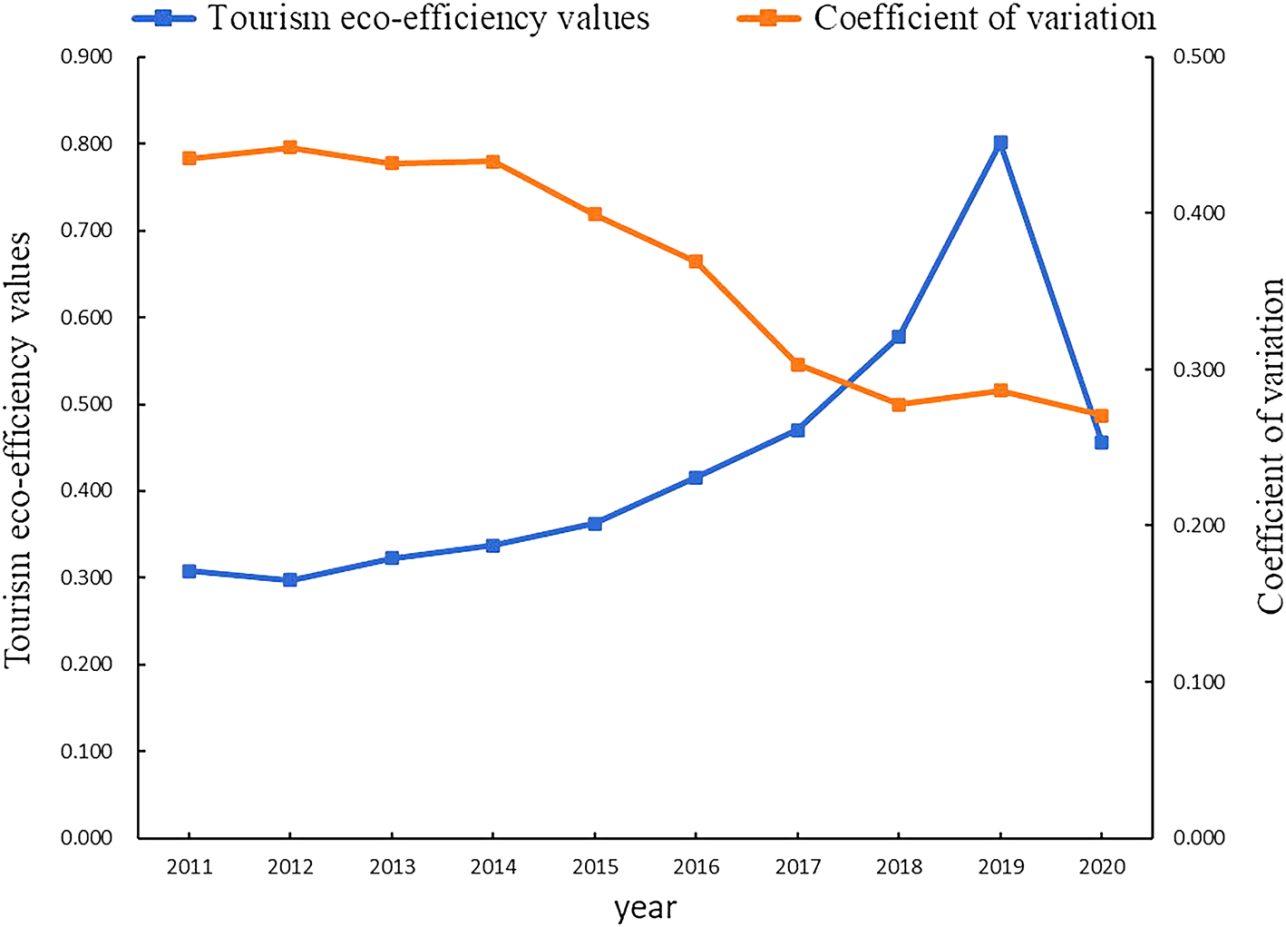
Tourism eco-efficiency values and coefficient of variation.

As can be seen from Fig. 2 and Table 2, the overall trend of tourism eco-efficiency in Fujian Province is fluctuating and increasing. The average value of tourism eco-efficiency in Fujian Province from 2011 to 2020 is 0.435, which indicates that the current tourism economic growth mode in Fujian Province shows a sloppy development, and the level of tourism eco-efficiency still needs to be improved. The average value of tourism eco-efficiency in Fujian Province increased from 0.308 in 2011 to 0.801 in 2019, an increase of 160.065%. However, in 2020, due to the impact by the COVID-19 pandemic, the total number of tourists dropped tremendously, the tourism economy was severely damaged, and the overall tourism eco-efficiency in Fujian Province showed a downward trend. In addition, the coefficient of variation of tourism eco-efficiency in Fujian Province shows a fluctuating decreasing trend, decreasing from 0.435 in 2011 to 0.271 in 2020, a decrease of 37.701%, reflecting that the difference in tourism eco-efficiency between cities in Fujian Province gradually decreased.

**Table 2:** Tourism eco-efficiency values.

City	2011	2012	2013	2014	2015	2016	2017	2018	2019	2020	Average value	Rank
Fuzhou	0.319	0.238	0.24	0.257	0.291	0.384	0.415	0.562	1.037	0.396	0.414	4
Xiamen	0.527	0.541	0.61	0.634	0.622	0.716	0.77	0.882	1.102	0.606	0.701	1
Putian	0.209	0.21	0.232	0.224	0.229	0.29	0.38	0.446	0.557	0.322	0.31	9
Sanming	0.196	0.205	0.234	0.262	0.289	0.325	0.377	0.481	0.631	0.395	0.34	7
Quanzhou	0.301	0.32	0.396	0.425	0.461	0.421	0.41	0.493	0.654	0.403	0.428	3
Zhangzhou	0.16	0.199	0.205	0.222	0.249	0.296	0.357	0.423	0.608	0.432	0.315	8
Nanping	0.525	0.494	0.469	0.488	0.548	0.628	0.651	0.811	1.147	0.715	0.648	2
Longyan	0.257	0.267	0.285	0.293	0.324	0.366	0.456	0.565	0.745	0.408	0.396	5
Ningde	0.272	0.202	0.233	0.228	0.248	0.311	0.415	0.542	0.732	0.42	0.36	6
Average value	0.308	0.297	0.323	0.337	0.362	0.415	0.47	0.578	0.801	0.455	0.435	
Coefficient of variation	0.435	0.442	0.432	0.433	0.4	0.369	0.303	0.278	0.287	0.271		

In terms of time periods, the development level of tourism eco-efficiency can be divided into three periods. First, the initial development period (2011–2014), the efficiency level of the cities in this period fluctuates greatly, the overall average value is only 0.316, showing the characteristics of “low quality and low efficiency”. The main reason is that under the background of “mass tourism” in this period, the development of the tourism industry is relatively crude, and there are problems such as the blind introduction of high-pollution tourism projects and disorderly development of tourism scenic spots in some areas of tourism industry development. Second, the high-quality transition period (2015–2019), during which the efficiency level showed an obvious upward trend. In 2016, Fujian Province became the first ecological civilization pilot zone in China and carried out a series of reform experiments about ecological civilization, and the effectiveness of energy conservation and emission reduction, optimization of resource allocation, and technological progress gradually emerged, which not only improved the economic efficiency of the tourism industry but also reduced the tourism development of environmental costs of tourism development, promoting the development of tourism industry in Fujian Province to become more environmentally friendly. Third, the decline period (2020), affected by the COVID-19 pandemic, tourism eco-efficiency declines in 2020, which is mainly due to the decline in tourism economic output and stagnation of tourism industry development caused by public health emergencies, which in turn led to a decline in tourism eco-efficiency.

In terms of regions, tourism eco-efficiency shows a fluctuating growth trend in all cities. The average efficiency of Xiamen, Nanping, Quanzhou, and Fuzhou ranked in the top four, while the average efficiency of other regions was lower than 0.4. Putian, with the lowest average efficiency, was 0.391 lower than Xiamen, which had the highest average efficiency, and the latter was 2.262 times higher than the former. It is worth noting that the efficiency values of Xiamen and Nanping always maintain a leading position and are much higher than other regions. The possible reason is that Xiamen belongs to the core area of economic, social, and ecological development in Fujian Province, the city is more mature and can invest a lot of capital in tourism ecological development, introduce high-quality talents, and continuously improve the efficiency of tourism energy use, thus promoting the coordinated development of tourism industry and ecological environment. Nanping's tourism resource endowment is in an advantageous position in Fujian Province, the tourism industry has gradually become the driving force of its economic development, and the development of the tourism industry tends to be mature, which can relieve the negative pressure brought by the ecological environment relatively quickly. However, the average efficiency of Putian, Zhangzhou, Sanming, and other regions is low, and the ecological development of tourism is to a certain extent constrained by the relatively sloppy way of tourism development, the lagging infrastructure construction such as transportation, and the insufficient investment in tourism ecological development funds and talents, which are the regions in Fujian Province that should focus on strengthening ecological tourism management.

### Spatial characteristics of tourism eco-efficiency

#### Spatial pattern evolution of tourism eco-efficiency

**Figure 3:**
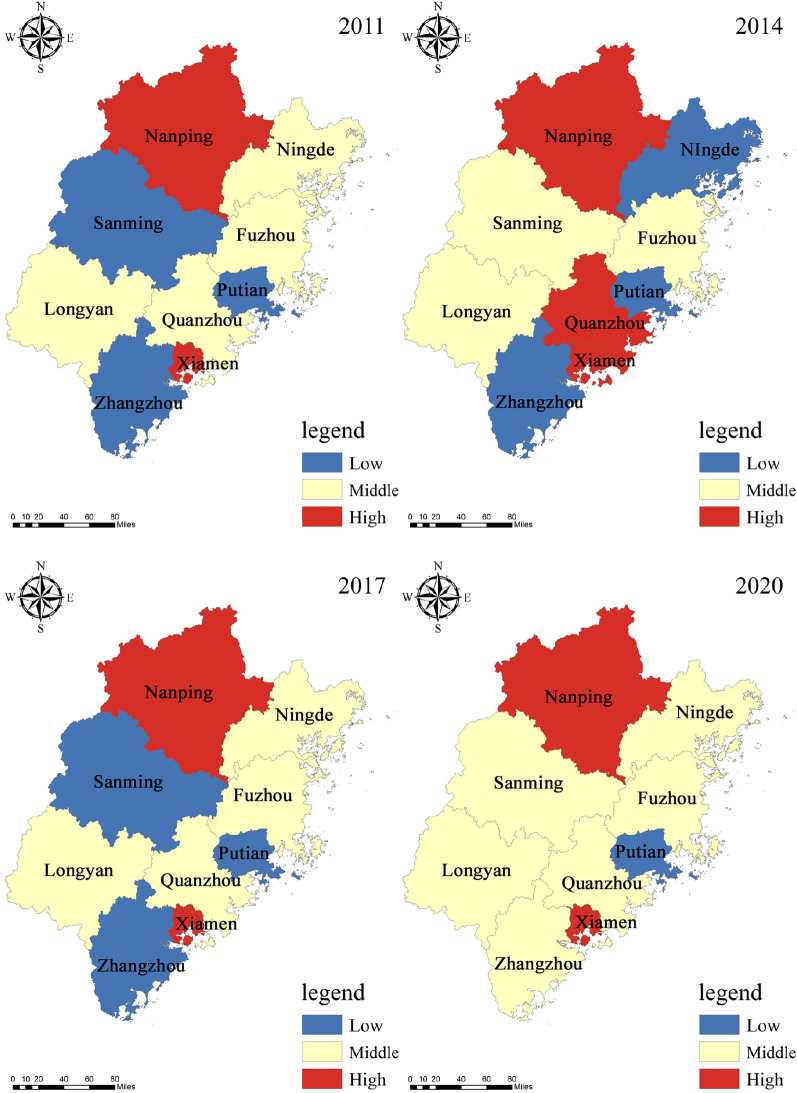
Spatial pattern of tourism eco-efficiency of Fujian province.

Selecting the intervals of the same 2011, 2014, 2017, and 2020 as time cross-sections, with the help of ArcGIS 10.8 software's natural fracture method into three categories: low level, medium level, and high level, to further clarify the spatio-temporal evolution characteristics of tourism eco-efficiency in Fujian Province municipalities and obtain the spatial pattern evolution map (Fig. 3).

Overall, the tourism eco-efficiency of Fujian Province has improved from 2011 to 2020, with fewer high-level areas and significant internal spatial differentiation. In 2011, the gap between individual cities is obvious, showing the phenomenon of inefficient spatial clustering and a smaller number of high-efficiency cities. Specifically, Xiamen and Nanping are located at high values, Fuzhou and Quanzhou at medium values and other cities at low values. The disadvantageous conditions of the economy, transportation, location, and technology in the early period, the advantages of tourism resources failed to be transformed into tourism economic advantages, coupled with the neglect of the balance between tourism economic development and ecological environment, resulting in low tourism eco-efficiency; In 2014, the spatial pattern remained unchanged, with urban efficiency levels mainly dominated by low and medium values. Nanping dropped from high to medium value, Fuzhou dropped from medium to low value, and the level in other regions remained unchanged. As the preliminary development of the tourism industry needs to invest a lot of labor, capital, and other elements to improve tourism infrastructure and develop urban tourism scenic spots, with the rapid expansion of industrial scale, the input elements of tourism industry are larger than the output elements, which will inevitably cause adverse impact on ecological environment and lead to high fluctuation of tourism eco-efficiency; In 2017, the overall efficiency level improved significantly, the number of cities with low values of efficiency level decreased significantly, and the degree of spatial divergence was reduced. Nanping improved from medium to high values, Sanming, Longyan, Ningde, Fuzhou, and Putian improved from low to medium values, and Zhangzhou remained in a low-efficiency state. Possible reasons for this are that Fujian Province became the first national demonstration zone for ecological civilization, and various places began to improve environmental protection policies and increase environmental protection treatment, increasing tourism eco-efficiency, but Zhangzhou is more slowly improved; In 2020, Zhangzhou increases from low to medium value, Putian decreases from medium to low value, other cities remain unchanged, and spatial divergence within Fujian Province still exists. Due to the impact of the Corona Virus Disease 2019, tourism eco-efficiency has declined to different degrees in both input and output values, especially reflected in the sharp decline in total tourism receipts, resulting in different degrees of changes in tourism eco-efficiency in each city.

#### Tourism eco-efficiency standard deviation ellipse and evolution of center of gravity.

**Figure 4:**
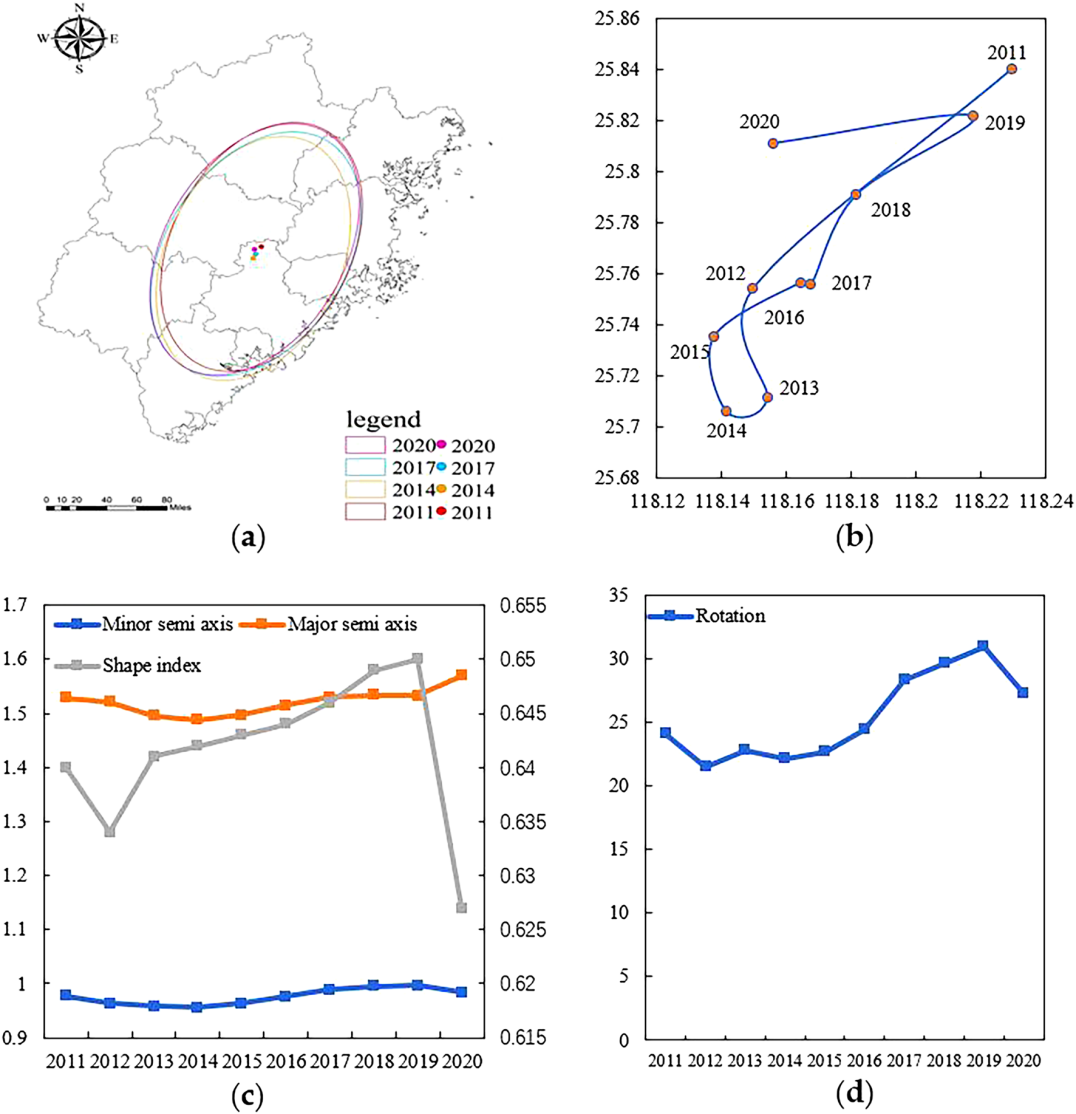
(**a**) Evolution of Standard deviation ellipse area; (**b**) Evolution of center of gravity; (**c**) Changes in major and minor axes and their ratios; (**d**) Rotation change.

The evolution of the space of tourism eco-efficiency in Fujian Province can be reflected by the change of standard deviation ellipse and center of gravity. As shown in Table 3 and Fig. 4, the standard deviation ellipse area from 2011 to 2014 is shrinking, and from 2014 to 2020 the standard deviation ellipse area keeps increasing (Fig. 4a), shrinking from 7.972 million km2 to 7.769 million km2, and then increasing to 8.126 million km2, indicating that the tourism eco-efficiency of Fujian Province is in an elevated trend, mainly because the cities in Fujian Province have paid more attention to green ecological development in recent years, and the tourism development model has been optimized, which has played a protective role for the ecological environment; From the shape index (Fig. 4c), the long half axis of the standard deviation ellipse is always larger than the short half axis, and the spatial distribution of tourism eco-efficiency in Fujian Province shows an obvious “northeast–southwest” directional distribution pattern. The shape index decreases in 2011–2012 and 2019–2020, indicating that the directionality of the spatial distribution of tourism eco-efficiency increases in this period and regional differences increase, while the shape index of 2012–2019 shows an increasing trend and the shape tends to be more and more positive circle, implying that the directionality of the spatial distribution gradually decreases in this period and the spatial agglomeration feature gradually becomes significant; From the trend of the long half axis of the ellipse, it shortens from 1.529 in 2011 to 1.488 in 2014, and then gradually increases until 1.569 in 2020, with an overall trend of continuous increase, indicates that the clustering of tourism eco-efficiency gradually decreases in the "northeast-southwest" direction. From the short half axis of the ellipse, it shrinks from 0.978 in 2011 to 0.956 in 2014, and then increases to 0.984 in 2020, showing an increasing trend, indicating that the tourism eco-efficiency in Fujian Province is expanding in both the long and short axis directions; From the change of azimuth angle (Fig. 4d), the azimuth angle increased from 24.104° in 2011 to 27.234° in 2020, with an overall increase of 3.130°, indicating that the spatial distribution pattern of tourism eco-efficiency in Fujian Province deflected from “northeast–southwest” to “east–west” by 3.130°, indicating that the tourism eco-efficiency in the northwest or southeast areas within the ellipse increased rapidly.

**Table 3:** Standard deviation ellipse parameters.

Year	Area (million/km2)	Minor semi axis (km)	Major semi axis (km)	Rotation (°)	Shape index
2011	7.972	0.978	1.529	24.104	0.640
2012	7.906	0.964	1.521	21.482	0.634
2013	7.797	0.958	1.495	22.800	0.641
2014	7.769	0.956	1.488	22.168	0.642
2015	7.821	0.963	1.497	22.704	0.643
2016	7.917	0.976	1.515	24.431	0.644
2017	8.004	0.989	1.530	28.315	0.646
2018	8.037	0.996	1.534	29.666	0.649
2019	8.037	0.997	1.533	30.931	0.650
2020	8.126	0.984	1.569	27.234	0.627

According to the results (Fig. 4b), the center of gravity of tourism eco-efficiency in Fujian Province is mainly located within the region of 25.68°N-25.84°N, 118.14°E-118.26°E, concentrated in the northern region of Quanzhou. In terms of the trajectory and direction of the center of gravity movement, the overall movement is to the southwest, and the study period is divided into three phases: 2011–2014, the center of gravity of tourism eco-efficiency moves to the southwest; 2014–2017, the center of gravity of tourism eco-efficiency moves to the northeast; 2017–2020, the center of gravity of tourism eco-efficiency moves to the northwest. As for the distance and rate of movement, only 2011–2012 and 2019–2020 have larger movement distances and rates, which indicates that the development of tourism eco-efficiency in Fujian Province tends to be stable during the study period.

### Analysis of the influencing factors of tourism eco-efficiency

Traditional geographic detectors are usually graded based on empirical knowledge, with less attention to discrete continuous variables and insufficient quantitative research, thus affecting the explanatory power of factors on tourism eco-efficiency. Therefore, this paper discrete six continuous-type influencing factors of economic development level (X1), urbanization level (X2), tourism industry structure (X3), transportation infrastructure input (X4), education level (X5), and technology innovation level (X6), and explored the influence of each driving factor on tourism eco-efficiency space in Fujian Province by using factor analysis and ecological analysis in the optimal parameters-based geographical detector model.

#### Selection of influencing factors

The evolution of the spatial pattern of tourism eco-efficiency in Fujian Province is a complex process, and in the process of evolving from the original spatial pattern to the existing pattern, it will be influenced by a combination of factors such as urban economy, transportation, resources, technology, etc. Following the relevant previous studies, the following influencing factors were selected: (1) Economic development level (X1). The development of the urban economy provides financial support and material security for the development of urban tourism, which is conducive to the introduction of advanced concepts and technologies for the tourism industry and more funds for environmental protection, thus effectively promoting the improvement of the regional ecological environment, as well as promoting the improvement of residents' tourism consumption capacity and the expansion of the tourism market scale, expressed using GDP per capita^36,37^. (2) Urbanization level (X2). The urbanization process, through the spatial concentration of population and industry, leads to the improvement and optimization of supporting infrastructure, promotes the expansion of the tourism industry scale and tourism consumption level, pushes the optimization of tourism industry structure, accumulation of tourism factors and tourism innovation overflow, and can provide resource elements such as capital, talent, technology and information for the improvement of tourism ecological environment, expressed by the proportion of the urban household population to the total population^29,38^. (3) Tourism industry structure (X3). Development economics believes that structural transformation can promote eco-efficiency and that a reasonable tourism industry structure is the key to tourism eco-efficiency. The optimization of the tourism industry structure is conducive to the healthy development of the tourism industry and thus has an impact on tourism eco-efficiency, which is expressed by the proportion of tourism revenue to GDP^39^. (4) Transportation infrastructure inputs (X4). Infrastructure is an important objective condition for the smooth development of tourism activities, and infrastructure inputs can better contribute to the optimization of resources at the existing level of technology and have a certain degree of impact on tourism eco-efficiency. Transportation conditions are one of the most important facilities in the infrastructure of the tourism industry and directly affect the willingness of tourists to enter the tourist destination. The improvement of transportation conditions has an important role in the gathering of tourists, capital, and technology in tourist places. Therefore, transportation conditions were chosen to measure the level of infrastructure investment, expressed as the ratio of road miles to land area^40^. (5) Education level (X5). A high level of education determines the development of industry, economy, and society. Higher education is an important part of training high-quality human resources, which to some extent reflects the level of innovation capacity of a region, and a high-quality workforce helps the tourism industry to develop in a green and low-carbon direction, thus contributing to the improvement of tourism eco-efficiency, expressed by the number of students enrolled in higher education institutions per 10,000 people^41,42^. (6) Technological innovation level (X6). According to economic growth theory, the fundamental driving force of long-term economic growth lies in technological progress. The application of innovative technologies in the tourism industry can reduce carbon emissions and energy consumption, improve the efficiency of tourism resources utilization, and reduce environmental pollution, thus achieving sustainable development of the tourism industry, expressed by the proportion of science and technology expenditures to public finance expenditures^43^.

#### Optimal parameter selection

**Figure 5:**
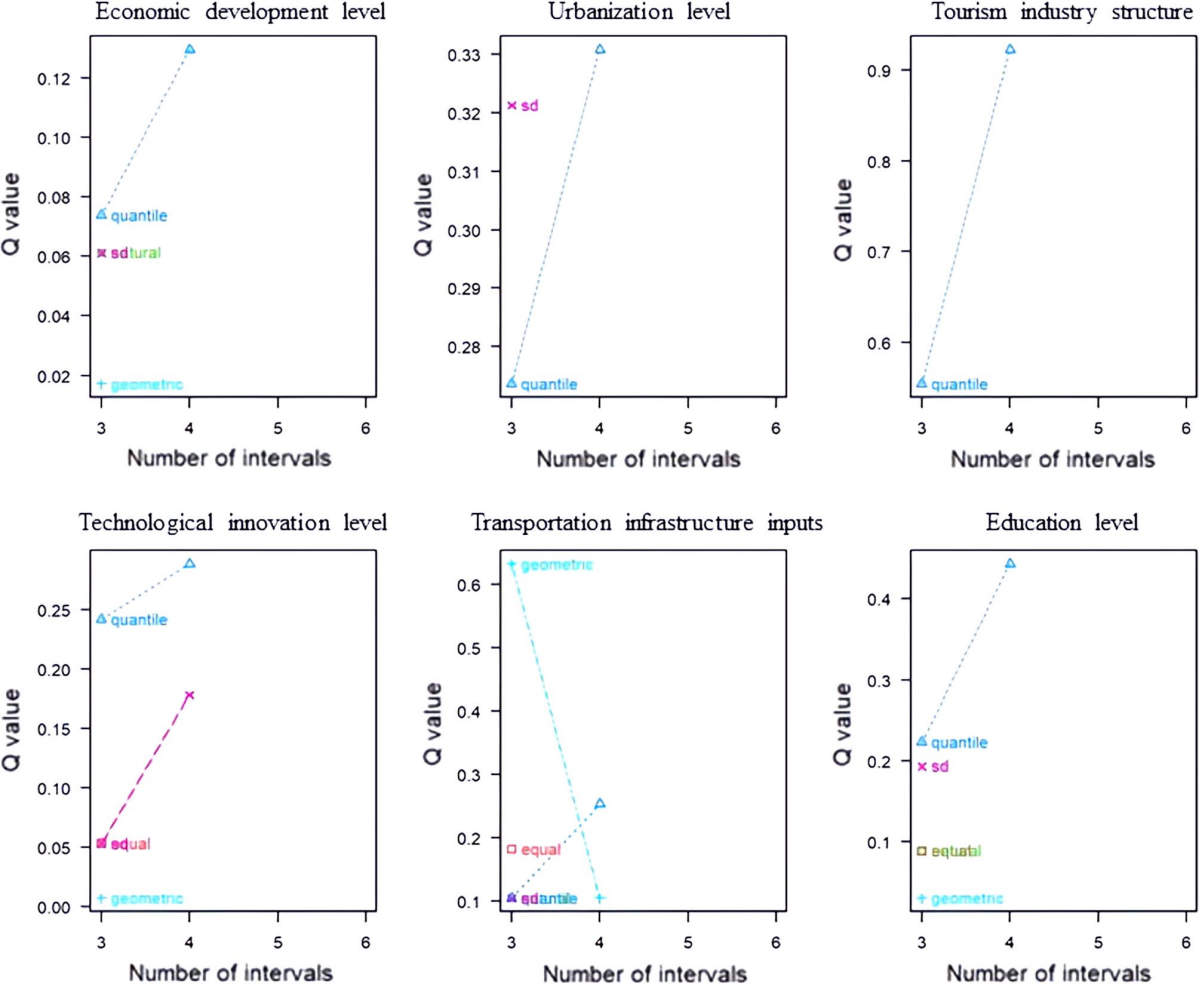
Selection of the optimal spatial data discretization method.

**Figure 6:**
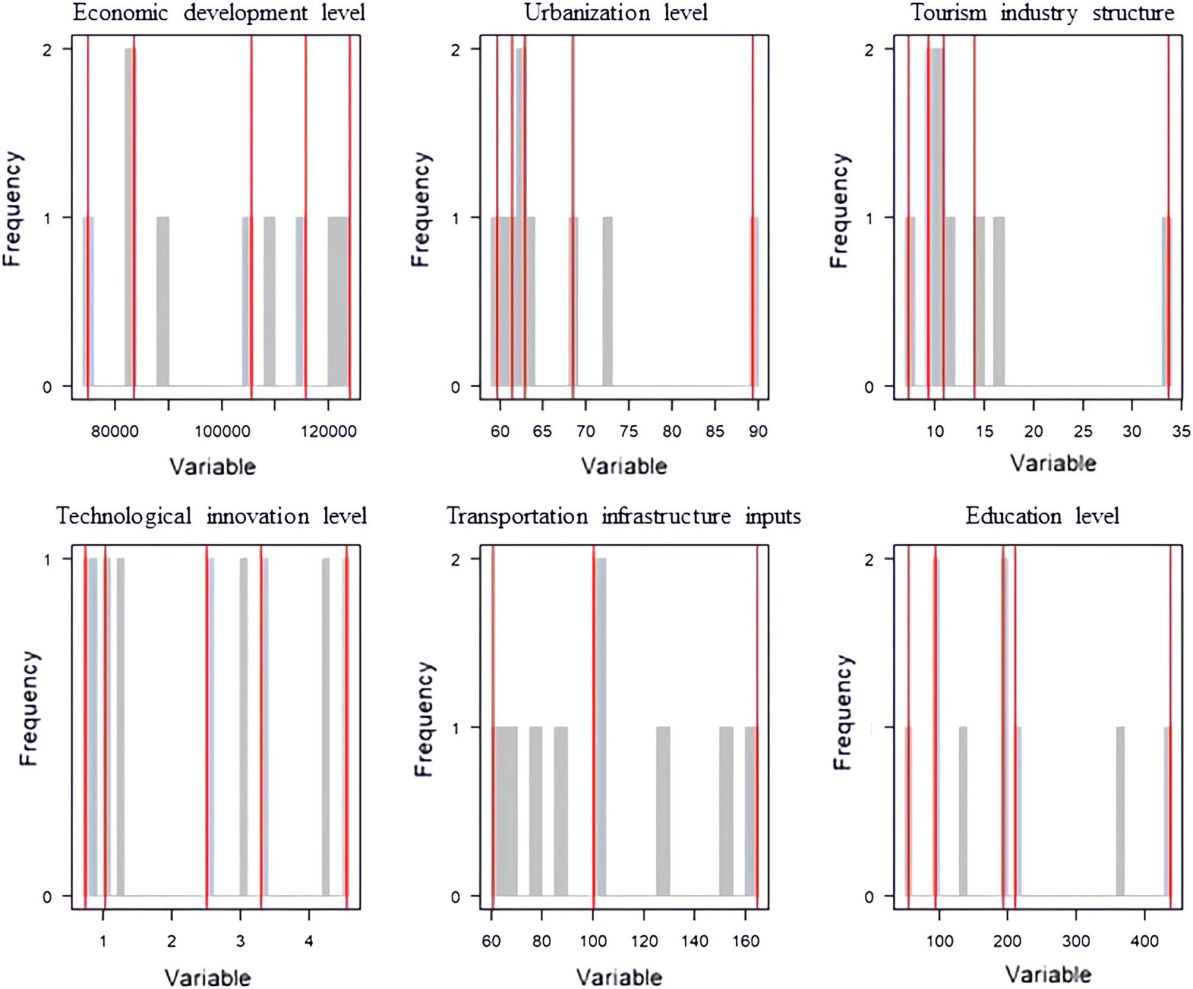
Optimal parameter breakpoint.

In the process of applying the geographic detector, the optimal discretization method and the number of combinations were screened, and the number of intervals was set to set to 3–6 classes (Fig. 5). Influence factors X1, X2, X3, X4, X5, and X6 all have the largest q-value in the quantile classification method and the largest q-value when the number of intervals is 4. Therefore, each of the 6 influence factors should be classified into 4 categories by quantile in the geographical detector as the optimal parameter selection (Fig. 6).

#### Analysis of factor detection results

As can be seen from Table 4, there are some differences in the influence of various influencing factors on tourism eco-efficiency in different years. The mean value of the influence of each influencing factor during the study period shows that the tourism industry structure (q = 0.893) has the greatest explanatory power, which is the core influencing factor. Education level (0.370), urbanization level (q = 0.350), technological innovation level (0.343), and transportation infrastructure input (0.305) all have strong explanatory power on the spatial variation of tourism eco-efficiency in Fujian Province, which is important influencing factors. The economic development level (0.240) always has a low explanatory power, which is a general influencing factor.

**Table 4:** Results of factor detection.

	X1	X2	X3	X4	X5	X6
2011	0.320	0.411	0.909	0.349	0.387	0.386
2014	0.221	0.246	0.801	0.289	0.295	0.270
2017	0.291	0.411	0.939	0.353	0.353	0.427
2020	0.130	0.331	0.923	0.253	0.443	0.289
Average value	0.240	0.350	0.893	0.311	0.370	0.343

Analysis of core influencing factors: tourism industry structure is the dominant factor affecting the spatial differentiation of tourism eco-efficiency in Fujian Province, indicating that a reasonable tourism industry structure is the key to improving tourism eco-efficiency. Through industrial structure optimization, the labor force and capital of the tourism industry are reconfigured between different sectors, flowing from sectors with high energy consumption and low productivity to sectors with low energy consumption and high productivity, which can effectively eliminate the damage and pollution of the ecological environment by tourism industry development.

Analysis of important influencing factors: the improvement of education level will help the cultivation of tourism talents, and high-quality talents will help the tourism industry to reduce the loss rate of tourism resources, improve the allocation efficiency of production factors, adopt advanced technology willingness, and provide eco-tourism products or services. In addition, the improvement of education level will improve the awareness of environmental protection of regional residents to a certain extent, and the way of travel of residents tends to be green and low-carbon, thus contributing to the improvement of tourism eco-efficiency; Urbanization is a process of population gathering and industry gathering. The expansion of urbanization scale and the improvement of quality promote the tourism industry to gather in specific areas, which can provide resources such as funds, talents, technology, and information for the improvement of tourism ecological environment and help the tourism industry to develop ecologically; The improvement of the level of technological innovation has greatly changed the traditional operation and management of the tourism industry. Technological progress provides technical support for the ecological development of the tourism industry, which can enhance the ability of tourism enterprises to save energy and reduce emissions, improve the structure of energy consumption, increase the efficiency of the use of regional tourism energy resources, reduce operating costs, expand tourism economic output, and play an important role in improving tourism eco-efficiency; Infrastructure investment determines the convenience of tourists going to tourist destinations and is the foundation of the development of tourism industry in a region. Imperfect infrastructure such as public service facilities and transportation is difficult to meet the needs of tourists, which will directly affect tourists' choice and evaluation of tourist destinations and hinder the development of the tourism industry. Therefore, infrastructure upgrading has practical significance for improving tourism eco-efficiency.

Analysis of general influencing factors: the detection value of economic development level is relatively low, which indicates that tourism eco-efficiency in Fujian Province is less dependent on the level of economic development. The improvement of economic development level is conducive to the introduction of advanced ideas and technologies and more investment in environmental protection, but it also increases the use of tourism energy, which brings adverse effects to the environment and thus hinders the improvement of tourism eco-efficiency.

#### Analysis of ecological detection results

Ecological detection was used to investigate whether there was a significant difference in the influence of the 2 factors on the spatial differentiation of tourism eco-efficiency, with "Y" indicating significant relative importance between the two influencing factors and "N" indicating insignificant relative importance between the two influencing factors. From the results of the ecological detection (Table 5), there is no significant difference between the effects of X2 (urbanization level) and X1 (economic development level) on the spatial differentiation of tourism eco-efficiency compared to each other; Significant differences in the effects of X3 (tourism industry structure) compared to X1 (economic development level) and X2 (urbanization level) on the spatial differentiation of tourism eco-efficiency; There is a significant difference in the effect of X4 (transportation infrastructure investment) on the spatial differentiation of tourism eco-efficiency compared with X3 (tourism industry structure), and there is no significant difference in the effect on the spatial differentiation of tourism eco-efficiency compared with the two factors of X1 (level of economic development) and X2 (level of urbanization); There is a significant difference in the effect of X5 (education level) compared to X1 (economic development level) on the spatial differentiation of tourism eco-efficiency, and there is no significant difference with X2 (urbanization level), X3 (tourism industry structure) and X4 (transportation infrastructure input); There is a significant difference in the effect of X6 (technology innovation level) compared with X3 (tourism industry structure) on the spatial differentiation of tourism eco-efficiency, and there is no significant difference with the other four factors. The above results further prove that the tourism industry structure has an important influence on the spatial differentiation of tourism eco-efficiency, therefore, it is of great practical significance to study the regional tourism industry structure, and it also provides a referable direction for regional improvement of tourism eco-efficiency.

**Table 5:** Results of ecological detection.

	X1	X2	X3	X4	X5	X6
X1						
X2	N					
X3	Y	Y				
X4	N	N	Y			
X5	Y	N	N	N		
X6	N	N	Y	N	N	

## Conclusions

The study of tourism eco-efficiency assessment and the spatial pattern is an important basis for revealing the development quality and trend of the tourism industry in Fujian Province. The main conclusions are as follows.

Firstly, the overall trend of fluctuating growth of tourism eco-efficiency of each city in Fujian Province indicates that all cities in Fujian Province are paying more and more attention to improving the quality of the tourism industry. The regional differences in tourism eco-efficiency are significant, but the coefficient of variation is fluctuating and decreasing, indicating that the regional differences are gradually decreasing, and the development of the tourism industry and eco-environmental protection in Fujian Province have been developing towards a balanced direction in recent years. The mean values of tourism eco-efficiency in each region in descending order are: Xiamen, Nanping, Quanzhou, Fuzhou, Longyan, Ningde, Sanming, Zhangzhou and Putian. Secondly, from the evolution of spatial pattern, the spatial distribution pattern of tourism eco-efficiency in Fujian Province is more stable and shows the phenomenon that Xiamen and Nanping continue to lead. Thirdly, in terms of influencing factors, tourism eco-efficiency in Fujian Province is mainly affected by the structure of tourism industry. It is greatly influenced by education level, urbanization level, technology innovation level, and transportation infrastructure investment. It is worth noting that it is less affected by economic development level, in other words, the level of economic development would not theoretically be an obstacle to the growth of tourism eco-efficiency.

## Discussion

### Theoretical implications

Firstly, the study of tourism eco-efficiency from the carbon peaking and carbon neutral targets is in line with the connotation of China's emphasis on the construction of ecological civilization and helps to promote the high-quality development of the tourism industry. This study enriches and expands the research related to tourism eco-efficiency and the goal of carbon peaking and carbon neutrality, while promoting the cross-fertilization of tourism economics, tourism geography, and other marginal disciplines, providing certain academic references for the study of eco-efficiency in other industries.

Secondly, scholars point out that the measurement of tourism eco-efficiency should focus on unexpected outputs^44^, this study incorporates unexpected outputs into tourism eco-efficiency measurement and constructs a more scientific, systematic, and perfect tourism eco-efficiency evaluation index system. Meanwhile, previous studies mainly used the traditional DEA model^5,7,19,45^, and this study draws on the optimized Super-SBM model, which solves the problems that the traditional DEA model cannot effectively rank decision units as well as input–output slackness and undesired outputs. It has more accurate tourism eco-efficiency measurement accuracy.

Thirdly, the optimal discretization method and quantity combinations are screened in this study using an optimal parameters-based geographical detector model, which improves the explanatory power of tourism eco-efficiency impact factors^46,47^.

### Managerial implications

Firstly, Similarly to the previous research^7,28,45^, the study found that tourism eco-efficiency varied significantly among cities in Fujian Province, and the results of the study can provide a point of reference for the government of Fujian Province to carry out macro-regulation and promote the sustainable development of the tourism industry in Fujian Province. At the same time, it can also provide reference significance for other regions. Specifically, the government should combine macro-control with market allocation, strengthen regional cooperation and ties, promote the balance of tourism resources among cities, improve the matching of tourism infrastructure, as well as balance capital, talents, and policy support, and stabilize the convergence and coordinated development of tourism eco-efficiency among cities^48^.

Secondly, scholars^39,49^ believe that the level of economic development has a significant positive effect on tourism eco-efficiency. However, we find that tourism eco-efficiency in Fujian Province is weakly affected by the level of economic development, which is consistent with the results of Qian^36^. Meanwhile, scholars^7,29,50^ believe that the spatial heterogeneity of tourism eco-efficiency in each region is strong, so cities should propose targeted policy measures to improve tourism eco-efficiency according to their own situation. On the one hand, the government should optimize the formulation of policies according to the importance of the influencing factors. For example, the tourism industry structure is the most important influencing factor of tourism eco-efficiency in Fujian Province, and each city should focus on improving the tourism industry structure and reasonably allocating tourism factors. On the other hand, the government needs to promote the synergistic enhancement of each influencing factor to gradually upgrade the tourism industry from high energy consumption, high pollution, and low-income development model to a green cycle development model with low consumption, low pollution, and high income to effectively improve tourism eco-efficiency.

Finally, the spatial distribution of tourism eco-efficiency has a close relationship with tourism resource endowment^51–53^, Xiamen and Nanping continue to rank at the top in terms of tourism eco-efficiency, confirming that areas with better tourism resource endowment also have higher eco-efficiency in the tourism industry. Therefore, attention should be paid to enhancing the comprehensive strength of cities and creating new tourism formats, as well as providing the necessary capital, talent, and technology guarantees for tourism industry development to promote the high-quality development of the tourism industry.

### Limitations and future research

This paper provides an in-depth discussion on tourism eco-efficiency and its influencing factors in Fujian Province and provides a theoretical reference for the improvement of tourism eco-efficiency in Fujian Province and other regions. At the same time, there are some shortcomings in this study that need to be addressed. First, due to the imperfection of China's tourism environmental monitoring system, this paper uses the "bottom-up" method to estimate tourism carbon emissions and tourism energy consumption indirectly, which has some limitations, subsequent studies can optimize the calculation method when the relevant monitoring system is improved. Secondly, the selection of factors influencing tourism eco-efficiency is still insufficient, In the future, we can combine the theories and methods of ecological, social and economic management disciplines to achieve interdisciplinary analysis to establish a more comprehensive and integrated index system.

### Supplementary Information

Supplementary Information.

## Data Availability

The datasets used and/or analysed during the current study available from the corresponding author on reasonable request.
